# Nutrition Smoothing: Can Proximity to Towns and Cities Protect Rural Children against Seasonal Variation in Agroclimatic Conditions at Birth?

**DOI:** 10.1371/journal.pone.0168759

**Published:** 2017-01-03

**Authors:** Amelia F. Darrouzet-Nardi, William A. Masters

**Affiliations:** 1 Department of Global Health Studies, Allegheny College, Meadville, Pennsylvania, United States of America; 2 Friedman School of Nutrition Science and Policy, Tufts University, Boston, Massachusetts, United States of America; TNO, NETHERLANDS

## Abstract

A large literature links early-life environmental shocks to later outcomes. This paper uses seasonal variation across the Democratic Republic of the Congo to test for *nutrition smoothing*, defined here as attaining similar height, weight and mortality outcomes despite different agroclimatic conditions at birth. We find that gaps between siblings and neighbors born at different times of year are larger in more remote rural areas, farther from the equator where there are greater seasonal differences in rainfall and temperature. For those born at adverse times in places with pronounced seasonality, the gains associated with above-median proximity to nearby towns are similar to rising one quintile in the national distribution of household wealth for mortality, and two quintiles for attained height. Smoothing of outcomes could involve a variety of mechanisms to be addressed in future work, including access to food markets, health services, public assistance and temporary migration to achieve more uniform dietary intake, or less exposure and improved recovery from seasonal diseases.

## Introduction

We define *nutrition smoothing* as the ability of an individual, a household or a population to maintain stable nutrition and health outcomes, despite potentially adverse changes in local circumstances. Smoothing nutrition goes beyond maintaining food security and diet quality to a deeper level of resilience, including prevention and recovery from disease [1]. Measuring and comparing degrees of nutrition smoothing is a first step towards identifying *how* people achieve stable nutritional outcomes, which is likely to depend on initial conditions and the type of shock to be absorbed. Many different mechanisms could be involved, such as agricultural production and food purchases to smooth dietary intake, health care services to prevent and treat seasonal diseases, or sanitation and vector control to block seasonal disease transmission. This study aims to define and measure nutrition smoothing using only cross-sectional data on the child’s birth month and nutrition outcomes, their location in terms of seasonal variation in rainfall and temperature, and their distance to towns or cities. Future work using other kinds of data would be needed to investigate which urban resources account for this smoothing effect, and how the more remote rural households might achieve the same degree of smoothing obtained by those closer to towns and cities.

The outcomes of interest for this study are child mortality, height, and weight, which collectively are indicators of past nutrition and health conditions as well as predictors of future well-being. The setting is the Democratic Republic of Congo (henceforth DRC), one of the world’s poorest countries whose vast expanse generates a wide range of degrees of rural isolation, with differences in latitude north and south of the equator creating variation in the timing and severity of seasonal cycles. Since household location cannot be assigned experimentally, we use the randomness of a given child’s birth month to identify the causal effect of exposure to seasonal risk factors, in a spatial difference-in-differences approach. Spatial difference-in-differences has been used primarily in economic studies, for example to estimate the value of greening vacant urban land [2], or the effect of an invasive species on land values [3]. We use this method to estimate the effect of proximity to towns on the degree to which child health depends on seasonal circumstances, controlling for unobservable and observable risk factors.

### Proximity to towns and nutrition smoothing in remote areas

In DRC and other settings, infrastructure and other investments to improve rural households’ access to markets and public services are well known to increase average productivity and welfare [4]. As another example, proximity to nearby healthcare clinics was a key factor for utilization of healthcare services by families in Tanzania [5]. Proximity to town facilitates access to virtually all man-made resources, from health care and government services to markets for goods and services. In the DRC and other very low income settings, remote rural households are much more reliant on natural resources and attendant environmental shocks, including seasonal variation in agroclimatic conditions. We do not observe how urban resources are used by each household; we only know that they can access those resources more easily if they live closer to a town or city. Households could use the services of a nearby town or city to buy or sell goods, visit the health clinic, or seek solutions for a livestock disease or pest infestation. Here we control for average nutrition outcomes to identify whether amenities in towns facilitate smoothing as such, using the natural experiment created by birth timing and exposure to season fluctuations. This strategy builds on the large and growing literature using birth timing as a natural experiment, such as research in Indonesia testing whether a supplemental nutrition program protected children exposed to a financial crisis in 1997–98 [6].

### Study contribution

The main contribution of this study is to define and measure the concept of nutrition smoothing. A secondary contribution is to demonstrate that this can be done using purely cross-sectional data, through a spatial difference-in-differences approach. In effect we treat each region as a repeated cross-section, using randomness in the timing of conception to identify the causal effect of exposure to seasonal rainfall and temperature, and various robustness checks on our tests for effect modification associated with proximity to town. DRC has many unique features influencing the study design, but the concept of nutrition smoothing and our method to measure it may have broad applicability in other settings.

## Background and Motivation

In DRC, approximately 75% of the population doesn’t consume sufficient calories for a healthy and active life [7–9], and the country has some of the world’s highest rates of child stunting (45.8%), wasting (14%), and underweight (28.2%) [10]. These deprivations reflect both longstanding poverty and recent disruptions associated with a protracted civil war. The Food and Agriculture Organization (FAO) estimated that per-capita food supply declined from 2595 kcal per person per day in 1994 to 1833 kcal per person per day in 2009 [7]. Various other indicators are also worsening over time, in contrast to encouraging trends in neighboring countries [11–12]. As in most of Africa, the majority of DRC’s population is agricultural, and arable land per person or per agricultural worker have declined sharply in recent decades [7]. The volume and value of crops commonly grown in DRC, such as cassava, sugar cane, maize, and plantains has also been declining since 1997 [7], and the lack of infrastructure or markets and services ensures that many households cannot effectively smooth consumption or protect children against adverse health shocks.

### Environmental variability and child health

Variation in environmental conditions has opened countless opportunities to study the causes of differences in health and other child development outcomes [13–14]. The substantial and illuminating body of literature in this area uses severe shocks such as a drought, famine, or war, as well as milder and sometimes predictable variation in temperature or other conditions in early life [15–25], and often finds important long-term consequences for an individual’s risk of disease, attained height, and labor productivity [26–31]. A key feature of child development is its sensitivity to environmental shocks at critical ages and developmental periods [32–34].

Seasonal cycles in birth outcomes, child health, and farmer well-being has been observed around the world, including most recently in Brazil [35], and Indonesia [36]. Both Brazil and Indonesia are similar to DRC in terms of vast sizes, locations in relation to the equator, predominant ecosystems, and high proportions of people with agricultural livelihoods. Even though seasons are relatively predictable, Gambian children born at unhealthy times have systematically lower weight-for-age and height-for-age than others [37], and have increased risk of mortality as young adults [38]. Seasonal patterns of this type can be extremely robust, for example even after controlling for within-mother and within-community characteristics by comparing siblings and children residing in the same village [39].

The worst time to be born is an empirical question, and is likely to depend on the type of shock and the circumstances of the household. One of the few biological constraints is that total energy demands on the mother are typically greatest in the last trimester of pregnancy, at birth and while breastfeeding [40]. This could help explain why children born during lean seasons may be most disadvantaged, as the harm they experience just before conception and around 0, 12 and 24 months of age outweighs the benefits of favorable conditions in mid-pregnancy and around 6, 18 and 30 months of age.

### Proximity to towns and child health

The aim of this study is to test whether proximity to towns and cities helps rural households avoid differences in health outcomes associated with seasonal fluctuations in rainfall and temperature. We build on the rich body of literature investigating the relationships between proximity to towns, price volatility, and consumption smoothing [41–43], particularly the finding that expansion of railroads in India had a protective effect in maintaining real incomes and reducing mortality in the face of environmental shocks [44]. Households that rely on agriculture for income and food may be most susceptible to climate variation and most unable to smooth consumption across seasons [45–47]. Anthropological evidence from Peru suggests that these fluctuations may be greatest for the most isolated rural households [48], but in other settings such as Bangladesh, even city-dwellers may experience seasonal shifts in food security and child weight-for-age [49].

## Methods

Our analytical method is spatial difference-in-differences, across three dimensions. First, we use climate data to identify regions with and without seasonal fluctuations. Next, we use the randomness of birth timing to identify exposure to seasonal risk where it exists. Finally, we use remoteness of households to identify whether proximity to towns confers resilience for children born in those places at riskier times. We use continuous variables for diagnostic regressions and exploratory exercises, then aggregate observations into dichotomous categories, add mother and community fixed effects and conduct various robustness checks against our identification strategy to address concerns about endogeneity and correlated errors [50].

To address our research question, we merge spatial and temporal data on child health, household characteristics, roads, terrain, land cover, towns, and civil conflict incidents across DRC. The spatial units of observation are one degree by one degree grid cells, which avoids endogeneity problems that may arise with administratively-chosen boundaries [51]. Each one-degree grid cell is approximately 69 square miles in size, an area which varies only slightly in tropical areas like the DRC; distances between degrees of longitude and latitude are more variable closer to the poles.

Our main source of data on nutrition outcomes is the DRC’s nationally representative Demographic and Health Survey (DHS), which was conducted in 2007 and again in 2013. Data collection for the DHS complied with the Helsinki Declaration of 1983 on ethics in research on human subjects [52]. The heights and weights of a sub-sample of children for each of the survey rounds were measured for N = 2,931 children in 2007 and N = 5,504 children in 2013. We dropped observations for families that had moved in the previous 6 years (n = 4,060) to ensure that children’s location at the time of survey was also their location at the time of birth. Observations flagged by DHS for biologically implausible measurements (where the absolute value of HAZ or WHZ is greater than 5) were also dropped (n = 3,302).

Our results control for exposure to civil conflict, which was and remains widespread in the study area. Conflict data are from the Armed Conflict Location and Event Dataset (ACLED), which details specific incidents of civil insecurity between 1997 and the present day for DRC and other countries [53].

We also incorporate geocoded data on 160 major towns from the Multipurpose Africover Database on Environmental Resources [54], calculating the Euclidean distances from the centers of each DHS survey cluster to the centers of each nearest major town point location using ArcGIS 10.0 [55]. ‘Proximity,’ defined as inverse distance (km^-1^), enters as a measure of access to all kinds of markets and public services.

In DHS data, the coordinates of each survey cluster have been randomly displaced by up to two kilometers in any direction for urban areas, and up to five kilometers for rural areas, with one percent of all survey clusters randomly displaced by up to 10 kilometers [52]. This intentional measurement error is designed to maintain the anonymity of survey participants and their communities, resulting in non-differential misclassification bias which attenuates the magnitude and significance of effects associated with household location [56]. Results obtained with more accurate location data would be somewhat larger in absolute value with smaller standard errors, but the estimates are still consistent and provide a conservative lower bound on the effect sizes and significance that would be obtained without error [57].

### Identification strategy

The naturally occurring random variation we exploit is the child’s month of birth, and hence their exposure to seasonal differences in rainfall and temperature. Our nutrition smoothing hypothesis is that potentially adverse conditions have smaller effects on child heights, weights and survival in locations that are closer to towns and cities. We control for time-invariant unobservable attributes of the child’s family and community using mother fixed effects in the mortality regressions, and cluster fixed effects in the height and weight regressions. We thereby compare each child to their siblings (for mortality regressions) and neighbors (for height and weight regressions) who were born at other times in the same place. To address the potential for spatially correlated errors, we pool children by risk exposure into dichotomous groups based on birth timing, degree of seasonal variation in rainfall, and distance to the nearest town. This dichotomous triple-difference approach to cross-sectional data mirrors standard difference-in-difference approaches with panel data, in which the identifying assumption of parallel trends is strengthened by comparing two pooled periods [50] and tested against placebo specifications in which any observed effect would be an artifact of the method.

Our analytical approach is illustrated in Table 1, showing how each subsample is classified in terms of exposure to seasonal risk and the potentially protective effect of proximity to town.

**Table 1:** Spatial variation in exposure to seasons, birth timing and access to towns.

Analytical design and hypothesized effects over triple difference-in-differences(region x birth timing x access to town)								
Region has a distinct rainy season? (= farther from the equator)	Yes				No			
Child was born in or after rainy season? (= Jan-Jun if lat.<0, Jul-Dec otherwise)	Yes*		No		Yes		No	
Household is closer to town? (= distance to town in km)	Yes	No**	Yes	No	Yes	No	Yes	No
*Hypothesized status*:	*Vulnerable to seasonal variation*				*Not vulnerable to seasonal variation*			
	Protected*	Affected**	*Unexposed*		*No effect*			

Note: Asterisks indicate hypothesis of significantly worse child nutrition relative to other groups in the same row.

*: the identifying assumption is that birth timing occurs randomly between seasons (tested).

**: the identifying assumption is that seasonal risk factors would have been similar in the absence of towns (untestable).

As shown by the first two rows of Table 1, our first hypothesized effect is that, in regions with distinct seasons, being born in one half of the year is associated with worse outcomes than being born in the other half. Inferring a causal effect of seasons relies on randomness of the child's birth month. We tested that assumption and found no evidence that other correlates of heights and weights cause selection bias into births at times with adverse outcomes. A further robustness check comes from testing for birth timing effects only in regions farther from the equator, against the benchmark of birth timing closer the equator where there is much less seasonal fluctuation in rainfall and temperature. In those ‘placebo’ regions, any statistical significance of birth timing would be an artifact of our study design. Our main hypothesis, shown in the third row of Table 1, is that among children born in places and at times where they are vulnerable to seasonal risk, being closer to towns is associated with less harmful outcomes. Here, inferring a causal effect relies on the *a priori* “parallel trends” observation that seasonal variation in local rainfall and temperature is unrelated to the household’s proximity to town.

### Measuring exposure to seasons

To capture seasonality in the DRC context, we use the absolute value of latitude of each DHS cluster’s location. Locations closer to the equator have generally uniform temperature and rainfall throughout the year, while locations both north and south of the equator have a more pronounced dry “winter” season [58]. The country stretches from approximately +5 degrees north to about -14 degrees south of the equator. Demarcation lines for our data are chosen to divide the sample into two roughly equal halves, which occurs at +4 and -4 degrees of latitude. Thus, most of the surveyed households subject to seasonal fluctuations were in the southern hemisphere, where the drier winter season occurs around June-August. Less than 20 percent of our sample (13,841 of the 69,641 births) is in the northern hemisphere where the timing of seasons is shifted by six months so that winter occurs around December- February. To construct a single variable that indicates births in a given season, we defined “*rain months”* to be the calendar month for households located in the southern hemisphere, and shifted 6 months forward for households in the northern hemisphere. For example, children born in the calendar month of January were recorded as such if in the southern hemisphere, and that month was recorded as “June” for the few children born in the northern hemisphere. These birth months were then aggregated into birth seasons, capturing a child’s exposure to similar seasonal conditions anywhere in the country using a single variable. With constructing this proxy measure of exposure to seasons, we were concerned with variations that are entirely predictable, and yet people may have been unable to avoid their negative impact. One reason could be that so many factors move together: during the hungry or lean season, food supplies from the previous harvests dwindle, gainful employment may be more difficult to find, disease incidence often increases, and maternal labor time and calorie expenditure may rise [59–60].

### Controlling for age to avoid survey timing effects

DHS data are typically collected in waves at specific times of year. The data we use were primarily collected in June 2007 and in December of 2013, as detailed in the supporting information. So, children born in earlier months (e.g. in May and in November of the respective survey round years) were surveyed at a younger age than those born in later months (e.g. in July or January, respectively). Since height and weight z scores vary systematically with age, to avoid artifacts due to survey timing we follow [61] and control for age using a linear spline specification based on the average time path of stunting and wasting actually observed in our data. For HAZ, the piecewise linear controls have three splines with knots at 6 months and 22 months of age, and for WHZ we have two splines with one knot at 12 months of age. The number and location of these splines approximates the nonparametric relationship we observe in the DRC data, which is similar to the age effects found in other settings [33].

### Econometric specification

Our primary specification is an OLS regression with interaction terms to test for difference in differences, and mother or community fixed effects to absorb unobservable characteristics and compare siblings or neighbors born at different times of year. Standard errors are clustered at the community level, of which there are 300 in the 2007 survey and 540 in the 2013 survey, for a total of 840 locations.

There are three dependent variables of interest, indicated collectively by *Zi* on the left-hand sides of the equations: whether the child was alive at the time of the survey, the height-for-age z score (HAZ) and weight-for-height z score (WHZ). Each regression controls for age in months (*A*_*i*_), or time elapsed since birth in the case of mortality regressions, in piecewise linear form as described above, and for child sex (*S*_*i*_) defined as 1 = male. Birth season (as *BS*_*i*_) enters as a binary variable (with 1 = births occurring between January through June in the southern hemisphere and occurring between July through December in the northern hemisphere). The absolute value of latitude for each DHS cluster *j* is used to stratify the sample between children around the equator who face little seasonal variation, and those farther from the equator who experience a dry winter season. Household wealth (*H*_*i*_) enters as a categorical variable computed by DHS as quintiles of the national distribution, based on ownership of durable goods in the household. To control for civil conflict, we use a continuous measure (*C*_j_) defined as the number of conflict fatalities recorded in the child’s grid-cell from their conception over their lifetime to the survey date. The underlying civil conflict data span from 2001 to 2013 for every grid cell in the country.

Household proximity to the nearest major town enters as a binary indicator (*R*_j_) of whether the household is relatively remote, with 1 = household faces greater distance to travel to the nearest major town. The cut-off is 28.8km based on the median Euclidean distance in our sample. The *R*_*j*_ binary variable also enters in interaction with birth season to construct our difference-in-differences specification, where the estimated coefficients on that interaction ($A\hat{T}E_{ij}$) can be interpreted as the average treatment effect of household remoteness on child mortality, heights or weights, given their exposure to the seasonal risk (i.e. the average treatment effect *on the treated*). A negative estimated ATE would indicate that being located far from town limits households’ ability to protect their children from harm.

The reduced form econometric models are shown below in Eqs 1 and 2. These estimating equations could be derived from a typical health production function where health status at the time of survey is a function of current and lagged health inputs, as well as key environmental characteristics such as sanitation, disease exposure, and parents’ health and childcare knowledge. For our empirical purposes, the reduced form model is sufficient. The main pathway through which we expect birth season to affect the health production function is through adverse conditions such as low food supply or high rates of disease transmission, either of which could have affected a child directly or indirectly through the mother’s health during the sensitive periods of gestation and infancy. The subscript *i* indexes children, *k* indexes the linear age splines, and *j* indexes DHS clusters (household locations). *ε*_*i*_ is a stochastic error term with the usual properties, and *δ*_*fe*_ refers to mother or location fixed effects.

Eq 1 is a diagnostic regression using continuous variables and no interaction terms, estimated using Ordinary Least Squares (OLS). In this specification, the absolute value of latitude (*Lat*_*j*_) enters linearly and continuously as degrees, and household remoteness enters continuously as proximity to the nearest major town in km^-1^ (*P*_*j*_). Eq 2 is the spatial difference-in-differences specification, which pools observations into binary variables for the child’s location and birth timing. We split the sample by distance from the equator to construct a placebo region where no effect is expected, and estimate the models with mother and survey community fixed effects to account for time invariant regional factors omitted from the model. Robust standard errors are clustered by survey community to account for potential correlations among respondents in the same areas. Management of the spatial data was done in ArcGIS 10 [55], and econometric analysis was performed in Stata/MP Version 12 [62].

$$Z_{i}=\alpha +\sum_{k=1}^{n}\beta_{k}A_{k}+\gamma_{1}S_{i}+\gamma_{2}H_{i}+\gamma_{3}C_{j}+\gamma_{4}P_{j}+\gamma_{5}BS_{i}+\gamma_{6}Lat_{j}+\varepsilon_{ij}$$ (1)

$$Z_{i}=\alpha +\sum_{k=1}^{n}\beta_{k}A_{k}+\gamma_{1}S_{i}+\gamma_{2}H_{i}+\gamma_{3}C_{j}+\gamma_{4}R_{j}+\gamma_{5}BS_{i}+ATE_{ij}\left(BS_{i}R_{j}\right)+\delta_{fe}+\varepsilon_{ij}$$ (2)

## Results

Descriptive statistics are presented in Table 2, for the whole sample and for each sub-sample used in the regressions. There is some variation in means and standard deviations by group, with children in regions with a dry winter were particularly thin with mean WHZ scores around -0.5 versus -0.2 for children around the equator. Conflict events are more frequent or intense in locations closer to the equator.

**Table 2:** Descriptive statistics by birth timing and exposure to seasonal variation.

*Birth timing*:*Presence of seasons*:	*Jan*.*-JuneNoneN = 18*,*009*	*Jan*.*-JuneDry winterN = 18*,*973*	*July-Dec*.*NoneN = 16*,*724*	*July-Dec*.*Dry winterN = 15*,*935*	*All BirthsN = 69*,*641*
**Child status**					
Children Alive (%)	84.6%	84.5%	83.7%	85.2%	84.5%
HAZ	-1.51 (*1*.*68*)	-1.51 (*1*.*62*)	-1.61 (*1*.*92*)	-1.26 (*1*.*80*)	-1.47 (*1*.*86*)
WHZ	-0.31 (*1*.*25*)	-0.47 (*1*.*12*)	-0.24 (*1*.*41*)	-0.45 (*1*.*31*)	-0.38 (*1*.*33*)
Age (months)	28.24 (*17*.*57*)	28.00 (*17*.*29*)	29.70 (*17*.*10*)	29.88 (*16*.*69*)	29.16 (*16*.*53*)
Firstborn (%)	23.8%	24.9%	23.8%	23.5%	24.5%
Short interval (%)	28.2%	27.9%	26.1%	19.74%	25.6%
Boys (%)	50.5%	51.2%	50.4%	50.2%	50.6%
**Household**					
Wealth (quintile)	2.61 (*1*.*27*)	3.20 (1.46)	2.60 (*1*.*26*)	3.25 (*1*.*45*)	2.92 (*1*.*40*)
Proximity (km^-1^)	0.11 (*0*.*23*)	0.16 (*0*.*27*)	0.10 (*0*.*23*)	0.15 (*0*.*27*)	0.13 (*0*.*26*)
**Environment**					
Conflicts	108.72 (*716*.*5*)	15.03 (*65*.*7*)	93.52 (*596*.*8*)	15.95 (*69*.*7*)	31.28 (*66*.*9*)
Latitude (abs val)	1.91 (*1*.*36*)	6.14 (2.01)	1.98 (*1*.*17*)	5.99 (*2*.*02*)	4.31 (*2*.*64*)

Note: Data shown are means and standard deviations (in parentheses). Births labeled as January-June occurred in calendar months July-December for children born in the Northern hemisphere (N = 17,159). Conflicts are total number of fatalities during the child’s year of birth in the respondent’s 1-degree square grid-cell of residence.

Exploratory t-tests for differences in child mortality, heights and weights across groups are shown in Table 3, for effects of the child’s gender, remoteness, and birth season. Mean HAZ and WHZ is lower for boy children than for girls, and mean HAZ but not WHZ is lower in remote areas compared to other locations. Boys have higher mortality risk, as do children living in remote locations. Mean HAZ is also lower for children born during January-June in southern hemisphere locations (or July-December in the northern hemisphere) as opposed to births during the other half of the year.

**Table 3:** Two-sample T-tests with equal variances.

	*Alive*	*HAZ*	*WHZ*
***Gender***			
Girls	0.85	-1.31	-0.33
Boys	0.84	-1.48	-0.44
Difference	0.009	0.17	0.10
Pr(T>t)	0.00**	0.000**	0.00***
***Household Location***			
Not Remote	0.85	-1.26	-0.38
Remote	0.83	-1.53	-0.39
Difference	0.03	0.26	0.02
Pr(T>t)	0.00**	0.00***	0.28
***Birth season***			
Born Jan.-June	0.84	-1.50	-0.39
Born July-Dec.	0.84	-1.28	-0.38
Difference	-.001	0.22	0.01
Pr(T>t)	0.69	0.00***	0.28

Data shown are means of each outcome across groups;

* p < .10,

** p < .05,

*** p < .01.

**Fig 1:**
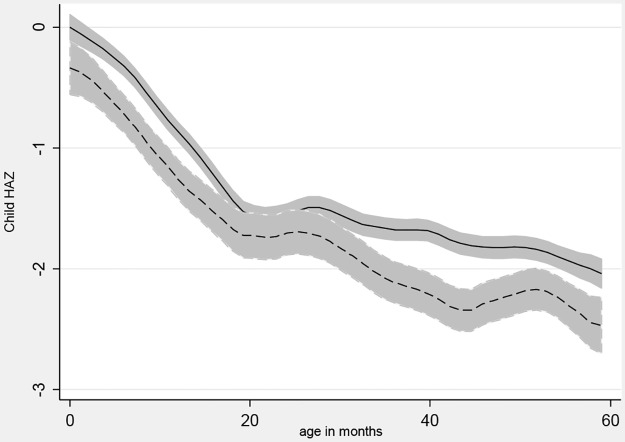
HAZ by child age and household remoteness. Notes: A local polynomial smoothing regression of child heights at each month of age, stratified by place of residence and the presence of seasons. Remote with seasons is dashed line. 95% Confidence Intervals included.

**Fig 2:**
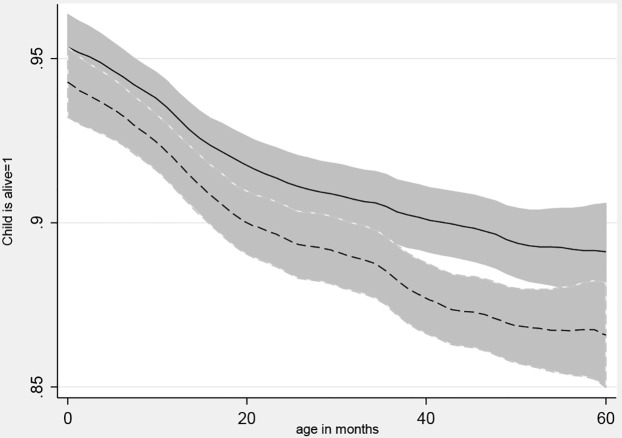
Child survival by child age and household remoteness. Notes: A local polynomial smoothing regression of whether the child is alive at each month of age, stratified by place of residence and the presence of seasons. Remote with seasons is dashed line. 95% Confidence Intervals included.

The onset and duration of stunting follow standard age patterns as shown in Fig 1, which uses Epanechnikov kernel-weighted local polynomial regressions to estimate mean HAZ values for each age in months, separately for remote households in areas with seasons versus the rest of the sample. Households in remote areas with seasons were hypothesized to have the worst outcomes, and Fig 1 shows that this group is indeed worse off than the rest of the sample. There is a steep decline in HAZ before 24 months of age, and then the slope flattens but is still negative. For WHZ, the decline ends at around 12 months of age with catch-up back to near zero by 5 years of age. Comparing remote versus other households, we see no significant differences at each month, although the HAZ path is consistently lower and the overall difference is significant as shown in Table 3. Fig 2 shows that children in remote areas are systematically more likely to have died, and this disparity increases with the time elapsed since birth.

**Fig 3:**
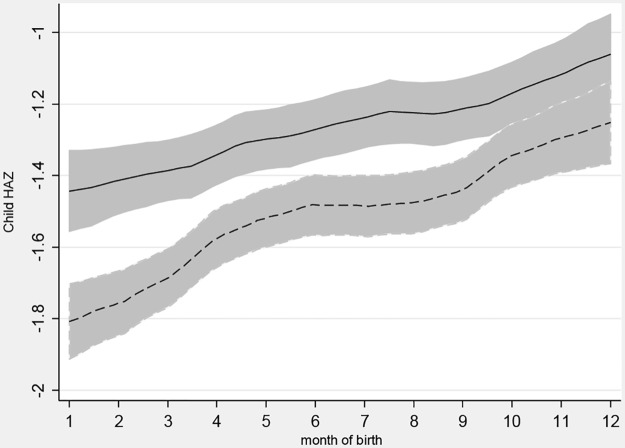
HAZ by month of birth and household remoteness. Notes: A local polynomial smoothing regression of child heights for births in each month of the year, stratified by place of residence and the presence of seasons. Remote with seasons is dashed line. 95% Confidence Intervals included. To account for inversion of seasons, birth date is shown by calendar month in the southern hemisphere, and for the northern hemisphere is shown as 1 = July, 2 = Aug. etc.

**Fig 4:**
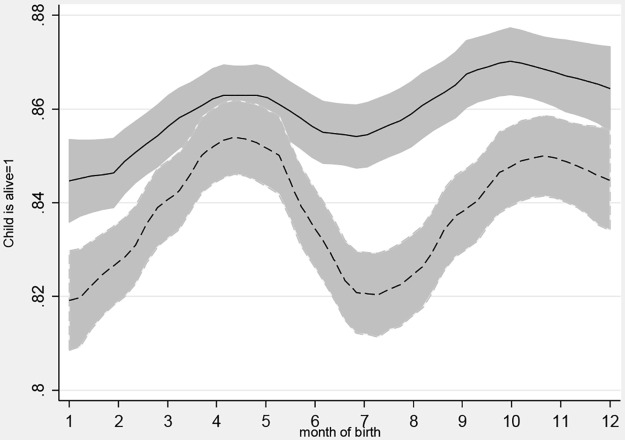
Child survival by month of birth and household remoteness. Notes: A local polynomial smoothing regression of whether the child is alive at the time of the survey for births in each month of the year, stratified by place of residence and the presence of seasons. Remote with seasons is dashed line. 95% Confidence Intervals included.

Variation in stunting and mortality by month of birth is shown in Figs 3 and 4, which like the previous charts use Epanechnikov kernel weighted local polynomial smoothing to estimate mean HAZ and mortality risk values for children born in each month, accounting for the inversion of seasons by hemisphere. Fig 3 reveals that the children born in July-December are systematically taller, and that children in remote areas are systematically shorter for each month of birth. Fig 4 shows that the fluctuations in mortality risk by month of birth had greater amplitude in remote areas with seasons, and that the children in remote areas with seasons are more likely to have died. These charts provide further evidence that the most disadvantaged group is those exposed to seasonal risk but not protected by a nearby town.

To address the relationship among all our variables, Table 4 presents results of a diagnostic OLS regression which estimates the association between children’s z scores and their age, sex, birth order, preceding birth interval, conflict exposure, household wealth, proximity to the nearest major town, and birth season. This exercise reveals the characteristic pattern that HAZ and WHZ both decline with age, although for HAZ the rate of decline is not significant for the first spline covering 0–6 months of age, and WHZ recovers significantly in the second spline after 12 months of age. Risk of death also decreases as more time since birth has elapsed. Male children have consistently lower z scores and higher risk of mortality. Conflict incidents in the grid cell of a child’s residence have statistically significant associations with nutritional outcomes: a negative association with HAZ and a positive association with WHZ. Firstborn children are more likely to have survived, and having a short preceding birth interval is associated with poorer height and survival outcomes. Conflict exposure is associated with an increased risk of death. Household wealth is positively associated with HAZ, but not WHZ. Wealth is also positively correlated with survival. Having controlled for these key factors, our variables of interest for the difference-in-difference design are not individually significant except in the mortality regressions. These exploratory regressions are informative, but for our main results we control for both observable and unobservable covariates using maternal or location fixed effects.

**Table 4:** Exploratory regressions with continuous explanatory variables.

		(1)	(2)	(3)
Variables	Unit/type	Child is alive Exploratory	HAZ Exploratory	WHZ Exploratory
Age spline 1	Linear spline	-0.017***	-0.074**	-0.107***
		(0.000)	(0.015)	(0.000)
Age spline 2	Linear spline	-0.002**	-0.072***	0.011***
		(0.015)	(0.000)	(0.000)
Age spline 3	Linear spline		-0.006	
			(0.104)	
Child is male	Binary	-0.115*	-0.133**	-0.108**
		(0.052)	(0.046)	(0.026)
Child is firstborn	Binary	-0.288***	0.021	-0.026
		(0.000)	(0.811)	(0.690)
Short preceding birth interval	Binary	-0.594***	-0.148*	-0.020
		(0.000)	(0.060)	(0.731)
Ln(fatalities during birth year)	Continuous	-0.062***	-0.114***	0.031**
		(0.000)	(0.000)	(0.032)
Household Wealth index	Categorical	0.145***	0.250***	0.053***
		(0.000)	(0.000)	(0.005)
Absolute value (latitude)	Continuous	-0.046***	-0.015	-0.017
		(0.000)	(0.313)	(0.130)
Proximity to town	km^-1^	0.281**	-0.022	0.162
		(0.045)	(0.878)	(0.137)
Born Jan.-June	Binary	0.134**	-0.107	0.075
		(0.024)	(0.114)	(0.126)
Constant	Constant	2.940***	-0.256	0.407***
		(0.000)	(0.200)	(0.003)
Observations	N	18845	3405	3473
*R*^2^	*R*^2^		0.179	0.073

The linear age splines are actually ‘time elapsed in months since birth’ for the mortality regressions.

Age splines control for child’s age at observation. Born Jan.-June is actually born July-Dec. in Northern hemisphere to account for inversion of seasons at the equator. Conflicts are the cumulative count nearby to the child’s cluster of residence during the child’s birth year. Errors clustered by DHS survey cluster (v001). *p*-values in parentheses;

* p < .10,

** p < .05,

*** p < .01.

Estimates from a pure difference-in-differences specification can be found in Table 5 below, where our main variable of interest is the triple interaction term indicating a child who was born during January-June, in a location with a dry winter, that is also relatively far from town. The estimated coefficient on this variable is statistically significant for heights, and not for weight or mortality.

**Table 5:** Triple difference-in-differences results for the pooled sample.

		(1)	(2)	(3)
Variable	Unit/type	Child is alive	HAZ	WHZ
Age spline 1	Linear spline	-0.016***	-0.080***	-0.100***
		(0.000)	(0.006)	(0.000)
Age spline 2	Linear spline	-0.002***	-0.067***	0.010***
		(0.001)	(0.000)	(0.000)
Age spline 3	Linear spline		-0.009***	
			(0.001)	
Short preceding birth interval	Binary	-0.510***	-0.187***	-0.039
		(0.000)	(0.002)	(0.387)
Child is male	Binary	-0.149***	-0.164***	-0.116***
		(0.001)	(0.002)	(0.003)
Ln(fatalities during birth year)	Continuous	-0.057***	-0.087***	0.018
		(0.000)	(0.000)	(0.152)
Proximity to town	km^-1^	0.744***	0.369	0.144
		(0.003)	(0.127)	(0.418)
Born Jan.-June	Binary	0.080	-0.097	-0.022
		(0.279)	(0.281)	(0.743)
Absolute value(latitude)	Continuous	-0.004	0.045***	-0.019
		(0.783)	(0.009)	(0.138)
Born Jan.-June*Proximity	Interaction	0.104	0.877**	0.232
		(0.769)	(0.013)	(0.367)
Born Jan.-June*Abs(lat)	Interaction	-0.002	0.018	0.007
		(0.914)	(0.464)	(0.686)
Abs(lat)*Proximity	Interaction	-0.053	0.038	-0.014
		(0.247)	(0.480)	(0.728)
Born Jan.-June*Proximity*Abs(lat)	Interaction	-0.021	-0.201***	-0.000
		(0.730)	(0.006)	(0.996)
Constant	Constant	3.081***	0.200	0.627***
		(0.000)	(0.244)	(0.000)
Observations	N	18845	3405	3473
*R*^2^	R^2^		0.144	0.056

The linear age splines are actually ‘time elapsed in months since birth’ for the mortality regressions.

Age splines control for child’s age at observation. Born Jan.-June is actually born July-Dec. in Northern hemisphere to account for inversion of seasons at the equator. Conflicts are the cumulative count in the child’s cluster of residence during the child’s birth year. Errors clustered by DHS survey cluster (v001). *p*-values in parentheses;

* p < .10,

** p < .05,

*** p < .01.

Results of our preferred difference-in-difference specification (Eq 2) are shown in Table 6. Following the research design described in Table 1, this test splits the sample into areas of interest with a dry winter season (columns 1 and 3) and the placebo regions with less seasonal variation in rainfall and temperature (columns 2 and 4). Each regression then includes interaction terms between season of birth and remoteness, where both are specified as binary variables. Regressions include either fixed effects for mothers (for mortality regressions), or for communities (for height and weight regressions), and standard errors clustered by survey site. Age profiles for HAZ are similar to the diagnostic regression and similar in the two regions. Gender differences for mortality, HAZ, and WHZ are also similar to the diagnostic regression and across the two regions. Interestingly, in this specification children are taller where there are more reported conflicts, but only in the areas without a dry winter. In this final specification, wealth and other controls are omitted due to their collinearity with the fixed effects, which absorb both observable and unobservable covariates.

**Table 6:** Preferred difference-in-differences results, splitting the sample by presence of seasons.

		(1)	(2)	(3)	(4)	(5)	(6)
Variable	Unit/type	AliveSeasons	AliveNo Seasons	HAZSeasons	HAZNo Seasons	WHZSeasons	WHZNo Seasons
Age spline 1	Spline	-0.021***	-0.022***	-0.051	-0.135***	-0.098***	-0.101***
		(0.000)	(0.000)	(0.220)	(0.003)	(0.000)	(0.000)
Age spline 2	Spline	-0.003***	-0.002***	-0.086***	-0.090***	0.010***	0.012***
		(0.000)	(0.000)	(0.000)	(0.000)	(0.000)	(0.000)
Age spline 3	Spline			-0.005	-0.003		
				(0.110)	(0.254)		
Short interval	Binary	-0.284***	-0.302***	-0.385***	-0.449***	-0.172***	-0.062
		(0.000)	(0.000)	(0.000)	(0.000)	(0.001)	(0.244)
Male	Binary	-0.117***	-0.126***	-0.029	-0.293***	-0.104*	-0.038
		(0.001)	(0.000)	(0.687)	(0.000)	(0.069)	(0.457)
Conflict exposed	Binary	-0.043	0.036	0.139	0.249**	-0.074	-0.062
		(0.399)	(0.547)	(0.148)	(0.038)	(0.274)	(0.509)
Jan.-June	Binary	-0.127**	0.079	-0.097	0.063	0.051	-0.093
		(0.011)	(0.210)	(0.210)	(0.573)	(0.521)	(0.355)
Jan.-June*Remote	Interaction	0.128*	-0.025	-0.329**	-0.188	-0.034	0.132
		(0.092)	(0.747)	(0.018)	(0.177)	(0.759)	(0.263)
Constant	Constant			0.158	0.537**	0.524***	0.624***
				(0.417)	(0.020)	(0.000)	(0.000)
Observations	N	17217	17297	4224	4211	4312	4319
*R*^2^	*R*^2^			0.290	0.299	0.083	0.077

The linear age splines are actually ‘time elapsed in months since birth’ for the mortality regressions. Born Jan.-June is actually born July-Dec. in Northern hemisphere to account for inversion of seasons at the equator. Age splines control for child’s age at observation. Mortality regressions include mother fixed-effects. Identification is possible here while including mother and community fixed-effects by interacting proximity with each child’s individual birth season. Height and weight regressions include survey cluster fixed-effects. Conflict exposure is a binary indicator of whether there was civil conflict in a 1-degree square of the child’s residence during the child’s year of birth. Errors clustered by DHS-cluster (v001). *p*-values in parentheses;

* p < .10,

** p < .05,

*** p < .01.

The average treatment effect of living farther from town (remoteness) when exposed to seasons is the estimated coefficient on the interaction term between them. Looking first at the “treatment” regions (columns 1, 3, and 5), the average treatment effect of household remoteness is statistically significant for survival and heights, but only in the locations with a dry winter season. The average treatment effect of remoteness is not significant for weights as an outcome. The effects for survival and heights are quite large in magnitude, with the height effect being similar to jumping about two quintiles in household wealth, and the survival effect similar to jumping one quintile in household wealth.

### Robustness tests

To check the robustness of our results, we can look first within Table 6 at results in the placebo regions with less seasonal fluctuation in rainfall. Here, there were no statistically significant average treatment effect estimates for any of the outcomes. To address any additional limitations of our main result, we conducted a wide variety of other robustness tests, the results for which can be found in the supporting information. Results do not change whether including or excluding households which have lived in their survey location for fewer than 6 years at the time of the interview (N = 4,060). Results also do not change whether including or excluding households which took trip lasting more than 1 month during the 12 months preceding the interview date (N = 6,969). To assess the presence of multicollinearity, variance inflation factors (VIF) are all relatively low, ranging from 1.00–2.17 (Table A in S1 File). We also examined whether civil conflict followed seasonal patterns, since that coincident cycle could have threatened identification by birth season. Nonparametric test demonstrated that there is no apparent seasonality in the intensity of conflict in DRC (Figure A in S1 File).

Since the DHS data were not collected uniformly over time, children born in different months were measured at different ages, and have consequently different levels of z score. Data for the DHS surveys utilized here were collected mainly during June for the 2007 survey and during December for the 2013 survey (Tables B and C in S1 File). The consequences of age at measurement for identifying seasonal effects has been highlighted by [60], using a type of diagram that we reproduced for each of the DRC survey rounds (Figures B and C in S1 File). These diagrams show the average age of measured children who were born in each month, and their average HAZ score. Children born in July (January for the 2013 survey) were the oldest when surveyed, and they also had the lowest average HAZ scores. Children born in December were the youngest on average when surveyed, and therefore had the highest HAZ scores. This effect is controlled for in our regressions using age splines, as recommended by [60]. For an additional robustness test on survey timing we re-ran all regression models using only the June data, and that had no appreciable difference relative to the data from other months.

Perhaps the most important threat to our research design is nonrandom birth timing. The frequency of births rises in March, April and May then has a long trough in July through December. We do not know why the number of births rises in March, April and May. That pattern could stem from a rise in conceptions during the dry “winter” (June, July and August), or from seasonal patterns in miscarriage and neonatal mortality. The amplitude of the curve is slightly lessened when measuring by “*rain month*”, implying that socioeconomic factors involving calendar months may be more important than seasons.

We tested whether seasonality in birth month confounded our results by measuring the relationships between our binary season-of-birth variable against all the explanatory variables in our dataset. These results suggest that the potential effect of endogeneity of birth timing did not influencing our findings (Table D and Figure D in S1 File).

### Falsification tests

In addition to the placebo region built into our main result, we also test our design against a variety of placebo outcomes as in [63]. These are dependent variables with no plausible mechanism by which they could have been caused by our independent variables of interest, so significant correlations would be artifacts of the research design that might also have given rise to our main result. The specific placebos we used here were: mother’s education, mother’s height, father’s education, years that the household has lived in the interview location, the size of the household (number of people), and the altitude in meters of the household’s location. Each of these occurred before or independently of when the child was born, and is used here to test whether our main results in Table 6 could be artifacts of the research design.

**Fig 5:**
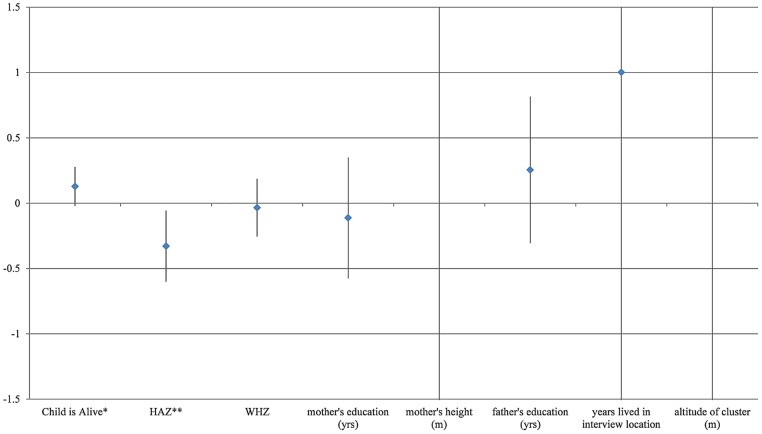
Falsification test results. Notes: In areas with seasons: estimated average treatment effects for various "placebo" dependent variables compared with Mortality, HAZ, and WHZ estimates. * indicates the ATE is significant at 10%, and ** 5%. Data shown are coefficient estimates (in blue) and 95% confidence intervals for “average treatment effects” in our preferred specification (Table 6), for our three dependent variables of interest followed by five ‘placebo’ variables for which no effect is expected of our ‘treatment’, due to the absence of any plausible mechanism of action.

Fig 5 below provides a visual comparison of our main results with the placebo variables. Each dot and bar shows the ATE point estimate with its 95 percent confidence interval, first for the main results and then for the seven placebo tests. The chart has been cropped to show coefficient estimates for effect sizes between -1.5 and +1.5, since the randomness around some of the placebos resulted in such wide error bars that our outcomes of interest could no longer be distinguished on the same chart. As it is, the chart shows that our precisely estimated negative effect on HAZ and WHZ is very different from the zero effects on any of the placebo outcomes. If we had found an effect of child’s birth season on any of the placebo outcomes, the validity of our identification strategy would have had to be questioned [64].

## Conclusions

This article exploits temporal and spatial differences in health risks to measure nutrition smoothing, defined here as the ability of a household or community to achieve similar outcomes for children exposed to different seasonal conditions. Our setting is the Democratic Republic of Congo (DRC), one of the world’s most impoverished countries whose vast expanse straddles the equator, exposing households to differing degrees of seasonal rainfall variation, and whose sparse urbanization and lack of infrastructure gives households very different distances to the nearest towns. In this context, we use naturally occurring randomness in the timing of conception and birth to construct a spatial difference-in-differences test of whether households with easier access to markets and public services can use that to protect their children from seasonal fluctuations in malnutrition and disease. Future work with other kinds of data could address which urban resources confer resilience to which seasonal risks.

An important feature of DRC is that children’s average health is not necessarily worst where seasonal variation is most extreme. For example, being geographically isolated could actually benefit households and children, as isolation and rugged terrain may protect them from violence or disease outbreaks in more populated areas [65]. Furthermore, being located near the equator may provide relatively uniform weather which is beneficial for growing crops, but also impose worse disease conditions because there is no interruption to the reproductive cycle of mosquitos [66]. Our research design takes account of these factors, building on the literature described above to isolate seasonal fluctuations from other factors and test for a protective effect of proximity to towns. Distance to urban resources may help explain why we sometimes observe a limited influence of income or wealth on health and nutrition outcomes [67]. As shown in this study, variation in environmental factors over time as well as space plays a relatively large role in those settings than in places with greater access to markets and services. The lack of nutrition smoothing also contributes to the extremely poor average outcomes observed in the DRC [51, 68].

Health outcomes in this study are child mortality, heights and weights at the time of the country’s 2007 and 2013 Demographic and Health Surveys (DHS). Our main result is that households’ proximity to towns and markets does protect children from seasonal fluctuations in health conditions at birth. For child height, the magnitude of gain from having above-median proximity to urban areas is similar in magnitude to the gain from being two quintiles higher in the local wealth distribution. Results of this magnitude are large but plausible, and help explain the rural-urban differences in health outcomes found in a wide variety of other settings. We subjected our findings to a variety of robustness tests, including comparisons of the estimated average treatment effect with similarly estimated coefficients in placebo regions and for placebo outcomes, selection bias in birth timing and child mortality, and other possible threats to identification.

Further work such as [69] would be needed to distinguish among the possible causal mechanisms involved, for example to distinguish between the role of private markets and the use of public services, and to identify the role of particular aspects of seasonal variation in dietary intake or disease burdens. Incorporating data on the amenities available in different towns would help distinguish which ones contribute most to nutrition smoothing in surrounding areas. Different mechanisms may be protective against different shocks, but all rely on infrastructure to link rural households with towns and cities where goods are traded and services are provided. These results add a new dimension to the role of rural infrastructure and access to towns. Interventions to lower households’ travel costs could help reduce their vulnerability, in addition to the many well-known investments that target specific causes of malnutrition such as improved diets, health care and reduced disease transmission.

## Supporting Information

S1 File **Table A: Variance inflation factors (VIF).** This table summarizes the variance inflation factors of key determinants of HAZ and WHZ in the final merged dataset. All variable definitions are as for Table 6. **Table B: Timing of data collection for 2007 survey.** Notes: This table enumerates the timing of data collection for the 2007 Demographic and Health Survey for the Democratic Republic of the Congo by month. **Table C: Timing of data collection for 2013 survey.** Notes: This table enumerates the timing of data collection for the 2013 Demographic and Health Survey for the Democratic Republic of the Congo by month. **Table D: Testing for endogeneity of birth timing, for whole sample and within climate zones.** This table shows results of a robustness test which measures any endogeneity of birth timing in the data. The dependent variable is a binary indicator of birth during the Jan.-June wet season. The regression was estimated using fixed-effects logit. All results include fixed effects for survey clusters (N = 840), with notation and variable definitions as in Table 6. p-values in parentheses; * p < .10, ** p < .05, *** p < .01. **Fig. A: Conflict incidents by month.** Notes: This figure was generated using the ACLED [60] for DRC. It aggregates the total count of conflict events by month across 16 years (1997–2013) in the country. **Fig. B: Mean age and HAZ at time of survey by calendar month of birth, 2007 DHS,** Notes: This figure is Fig 3 (pg. 39) of [70] reproduced for 2007 DRC data. The line shows average HAZ on the right axis by the child’s month of birth, and the bar shows their average age by month of birth on the left axis. As detailed in Tables 7 and 8, over three-quarters of the 2007 DRC surveys were implemented in June, and over three quarters of the 2013 DRC surveys were implemented in December. So, children born in July (for the 2007 round) and January (for the 2013 round) are surveyed at the oldest average age and have correspondingly lowest average HAZ scores. This ‘survey timing artifact’ effect is controlled for in our regressions using a flexible linear age spline, based on the time path of HAZ and WHZ scores shown in Figs 1 and 2. **Fig. C: Mean age and HAZ at time of survey by calendar month of birth, 2013 DHS.** Notes: This figure is Fig 3 (pg. 39) of [70] reproduced using the 2013 DRC data. The line shows average HAZ on the right axis by the child’s month of birth, and the bar shows their average age by month of birth on the left axis. As detailed in Tables 7 and 8, over three-quarters of the 2007 DRC surveys were implemented in June, and over three quarters of the 2013 DRC surveys were implemented in December. So, children born in July (for the 2007 round) and January (for the 2013 round) are surveyed at the oldest average age and have correspondingly lowest average HAZ scores. This ‘survey timing artifact’ effect is controlled for in our regressions using a flexible linear age spline, based on the time path of HAZ and WHZ scores shown in Figs 1 and 2. **Fig. D: Timing of births by calendar month and season.** Notes: Data shown are the number of children ever born in each month, as recorded across each DHS survey for DRC. The solid line refers to calendar months, and the dashed line uses a seasonal adjustment by hemisphere, where dates north of the equator are recorded as “January” for births in June, “February” for July, etc. In our regressions, these “rain months” are aggregated into six-month periods, since as children in higher latitudes who are born in the January-June period are more exposed to heavy rains and subsequently poor health outcomes than those born in the rest of the year. As shown here, more children were born in these adverse months than in July-December, as conception was slightly more likely to have occurred during the dry winter season. This pattern suggests that birth timing is either random or associated with factors other than variation in the child’s health prospects.
(DOCX)
