# Supplementary material for: Nutrition Smoothing: Can Proximity to Towns and Cities Protect Rural Children against Seasonal Variation in Agroclimatic Conditions at Birth?
Source: PLoS One. 2017 Jan 3;12(1):e0168759. doi: 10.1371/journal.pone.0168759 (PMC5207721; doi:10.1371/journal.pone.0168759)
Supplement: S1 File — Table A: Variance inflation factors (VIF). This table summarizes the variance inflation factors of key determinants of HAZ and WHZ in the final merged dataset. All variable definitions are as for Table 6. Table B: Timing of data collection for 2007 survey. Notes: This table enumerates the timing of data collection for the 2007 Demographic and Health Survey for the Democratic Republic of the Congo by month. Table C: Timing of data collection for 2013 survey. Notes: This table enumerates the timing of data collection for the 2013 Demographic and Health Survey for the Democratic Republic of the Congo by month. Table D: Testing for endogeneity of birth timing, for whole sample and within climate zones. This table shows results of a robustness test which measures any endogeneity of birth timing in the data. The dependent variable is a binary indicator of birth during the Jan.-June wet season. The regression was estimated using fixed-effects logit. All results include fixed effects for survey clusters (N = 840), with notation and variable definitions as in Table 6. p-values in parentheses; * p < .10, ** p < .05, *** p < .01. Fig. A: Conflict incidents by month. Notes: This figure was generated using the ACLED [60] for DRC. It aggregates the total count of conflict events by month across 16 years (1997–2013) in the country. Fig. B: Mean age and HAZ at time of survey by calendar month of birth, 2007 DHS, Notes: This figure is Fig 3 (pg. 39) of [70] reproduced for 2007 DRC data. The line shows average HAZ on the right axis by the child’s month of birth, and the bar shows their average age by month of birth on the left axis. As detailed in Tables 7 and 8, over three-quarters of the 2007 DRC surveys were implemented in June, and over three quarters of the 2013 DRC surveys were implemented in December. So, children born in July (for the 2007 round) and January (for the 2013 round) are surveyed at the oldest average age and have correspondingly lowest average HAZ scores. This ‘s [file pone.0168759.s001.docx]

Supporting Information (S1) File for:

### Nutrition smoothing: Can proximity to towns and cities protect rural children

### against seasonal variation in agroclimatic conditions at birth?

by Amelia F. Darrouzet-Nardi and William A. Masters

### S1 Table A: Variance inflation factors (VIF)

|  | *HAZ* | *WHZ* |
| --- | --- | --- |
| Age spline 1 | 2.17 | 1.33 |
| Age spline 2 | 1.61 | 1.37 |
| Age spline 3 | 1.5 | N/A |
| Child is male | 1.00 | 1.00 |
| Number of Conflicts | 1.25 | 1.25 |
| Wealth Quintile | 1.12 | 1.12 |
| Remote | 1.06 | 1.06 |
| Born Jan.-June | 1.04 | 1.01 |
| Abs(Latitude) | 1.19 | 1.19 |

Note: All results are as for Table 6.

### S1 Table B: Timing of data collection for 2007 survey

| Month | Number of surveys | Percentage (%) | Cumulative Percentage (%) |
| --- | --- | --- | --- |
| January | 23 | 0.08 | 0.08 |
| February | 1,935 | 6.98 | 7.07 |
| March | 128 | 0.46 | 7.53 |
| April | 826 | 2.98 | 10.51 |
| May | 3,172 | 11.45 | 21.96 |
| June | 21,166 | 76.40 | 98.35 |
| July | 453 | 1.64 | 99.99 |
| September | 3 | 0.01 | 100.00 |

Note: DHS administrative data for all child health variables.

**S1 Table C: Timing of data collection for 2013 survey**

| Month | Number of surveys | Percentage (%) | Cumulative Percentage (%) |
| --- | --- | --- | --- |
| August | 2,249 | 5.38 | 5.38 |
| September | 1,182 | 2.83 | 8.21 |
| October | 39 | 0.09 | 8.31 |
| November | 5,481 | 13.12 | 21.43 |
| December | 32,823 | 78.57 | 100.00 |

Note: DHS administrative data for all child health variables.

**S1 Table D: Testing for endogeneity of birth timing, for whole sample and within climate zones**

|  |  | (1) | (2) | (3) |
| --- | --- | --- | --- | --- |
| Variable | Units/type | Born Jan.-June | Born Jan.-June  Seasons | Born Jan.- June  No seasons |
|  |  |  |  |  |
| Child is Male | Binary | 0.009 | 0.023 | 0.005 |
|  |  | (0.762) | (0.632) | (0.895) |
|  |  |  |  |  |
| Wealth index | Categorical | -0.015 | -0.057 | 0.002 |
|  |  | (0.384) | (0.106) | (0.919) |
|  |  |  |  |  |
| Ln(fatalities) | Continuous | 0.014 | 0.003 | 0.018 |
|  |  | (0.125) | (0.830) | (0.152) |
|  |  |  |  |  |
| Proximity to town | km^-1^ | 0.319* | 0.538 | -0.047 |
|  |  | (0.069) | (0.227) | (0.875) |
|  |  |  |  |  |
| Abs val. (latitude) | Continuous | 0.021 |  |  |
|  |  | (0.138) |  |  |
| Observations |  | 18804 | 7060 | 11728 |

Note: Dependent variable is a binary indicator of birth during the Jan.-June wet season. Regression estimated using fixed-effects logit. All results include fixed effects for survey clusters (N=840), with notation and variable definitions as in Table 6. *p*-values in parentheses; * p<.10, ** p<.05, *** p<.01.

**S1 Fig. A: Conflict incidents by month**


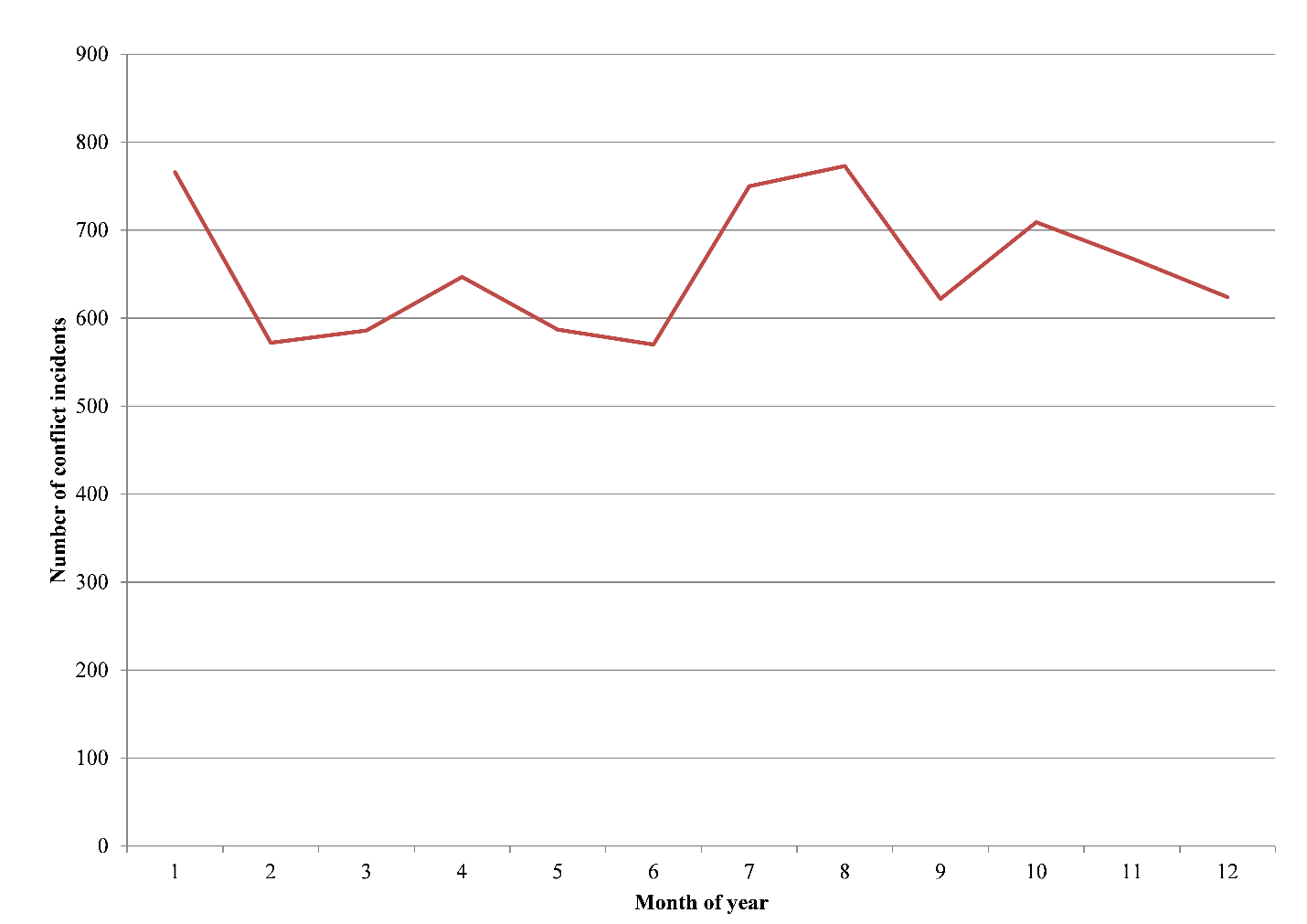


*Notes: This figure was generated using the ACLED [60] for DRC. It aggregates the total count of conflict events by month across 16 years (1997-2013) in the country.*

**S1 Fig. B: Mean age and HAZ at time of survey by calendar month of birth, 2007 DHS**


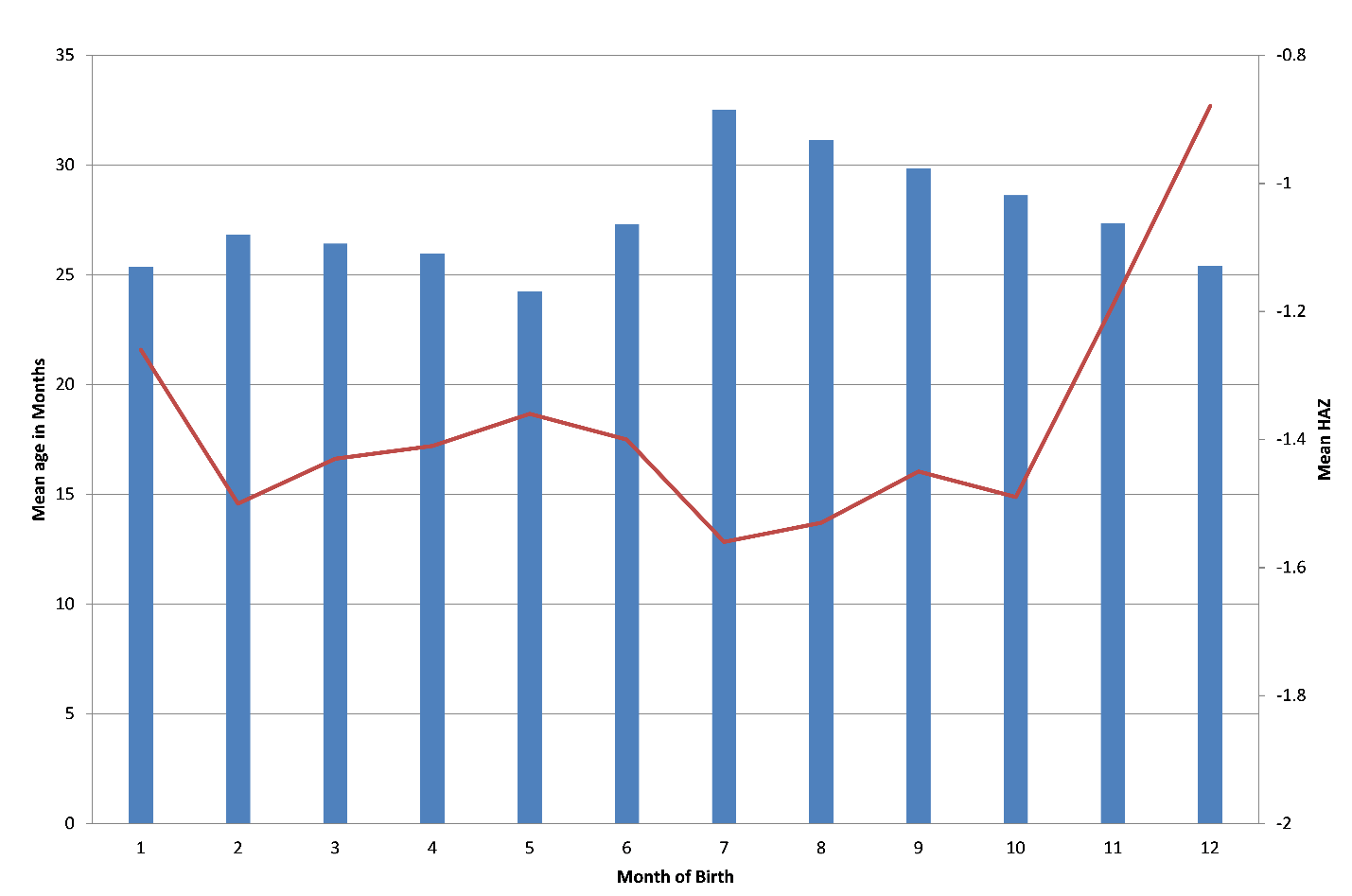


*Notes: This figure is Figure 3 (pg. 39) of [70] reproduced for 2007 DRC data. The line shows average HAZ on the right axis by the child’s month of birth, and the bar shows their average age by month of birth on the left axis. As detailed in tables 7 and 8, over three-quarters of the 2007 DRC surveys were implemented in June, and over three quarters of the 2013 DRC surveys were implemented in December. So, children born in July (for the 2007 round) and January (for the 2013 round) are surveyed at the oldest average age and have correspondingly lowest average HAZ scores. This ‘survey timing artifact’ effect is controlled for in our regressions using a flexible linear age spline, based on the time path of HAZ and WHZ scores shown in Figures 1 and 2.*

**S1 Fig. C: Mean age and HAZ at time of survey by calendar month of birth, 2013 DHS**


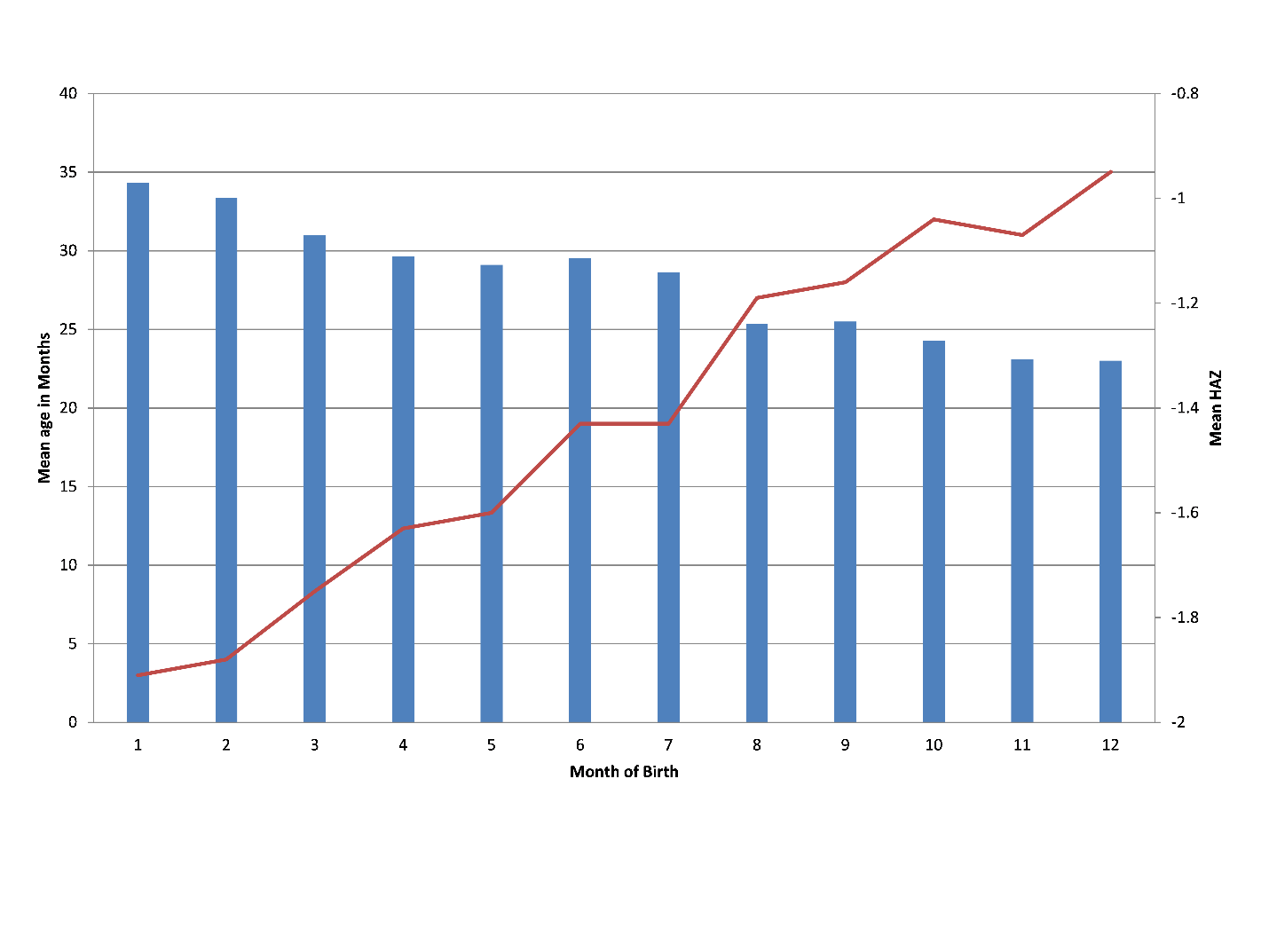


*Notes: This figure is Figure 3 (pg. 39) of [70] reproduced using the 2013 DRC data. The line shows average HAZ on the right axis by the child’s month of birth, and the bar shows their average age by month of birth on the left axis. As detailed in tables 7 and 8, over three-quarters of the 2007 DRC surveys were implemented in June, and over three quarters of the 2013 DRC surveys were implemented in December. So, children born in July (for the 2007 round) and January (for the 2013 round) are surveyed at the oldest average age and have correspondingly lowest average HAZ scores. This ‘survey timing artifact’ effect is controlled for in our regressions using a flexible linear age spline, based on the time path of HAZ and WHZ scores shown in Figures 1 and 2.*

**S1 Fig. D: Timing of births by calendar month and season**


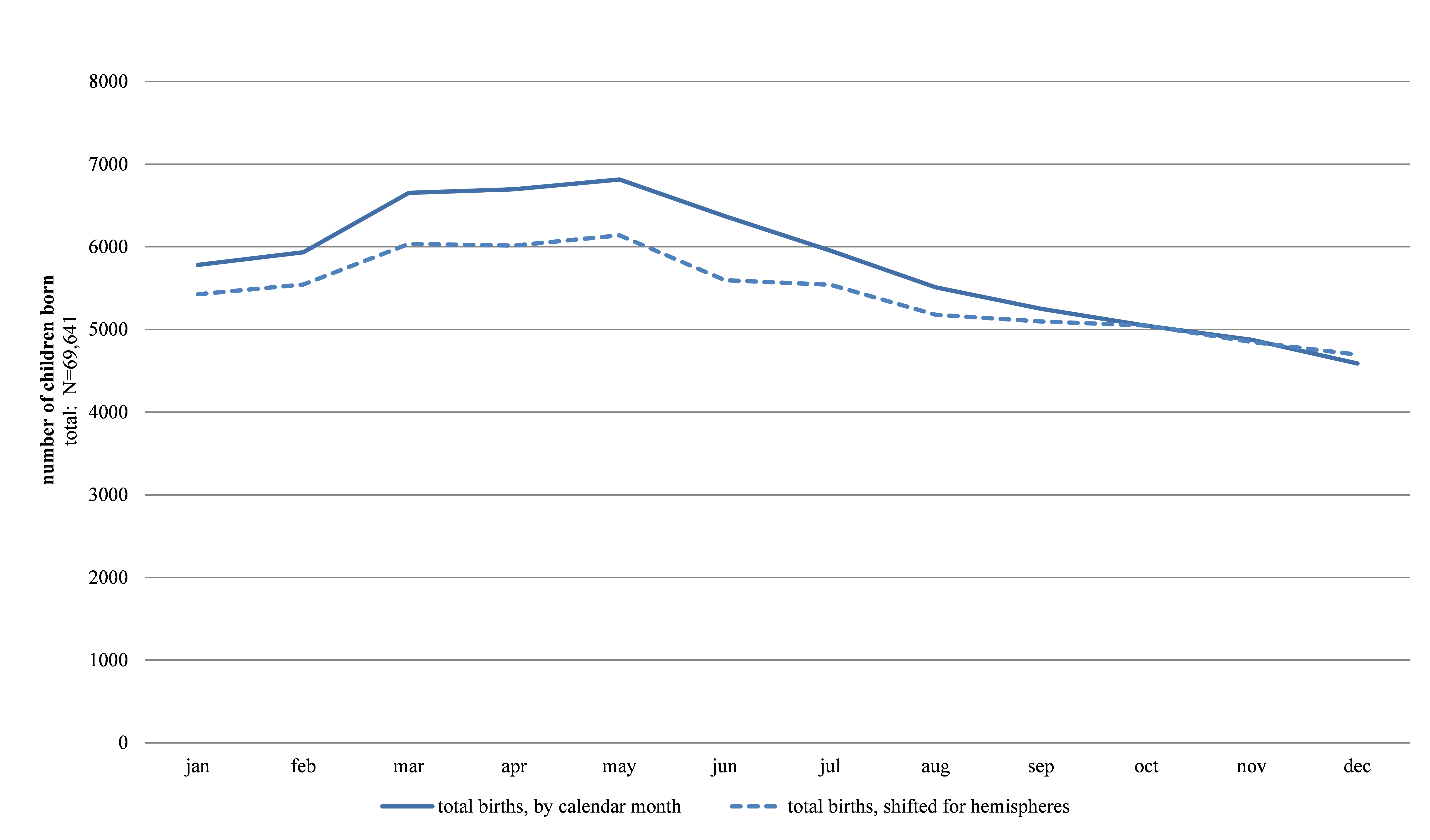


*Notes: Data shown are the number of children ever born in each month, as recorded across each DHS survey for DRC. The solid line refers to calendar months, and the dashed line uses a seasonal adjustment by hemisphere, where dates north of the equator are recorded as “January” for births in June, “February” for July, etc. In our regressions, these “rain months” are aggregated into six-month periods, since as children in higher latitudes who are born in the January-June period are more exposed to heavy rains and subsequently poor health outcomes than those born in the rest of the year. As shown here, more children were born in these adverse months than in July-December, as conception was slightly more likely to have occurred during the dry winter season. This pattern suggests that birth timing is either random or associated with factors other than variation in the child’s health prospects.*
